# Retraction: MicroRNA let-7c Inhibits Cell Proliferation and Induces Cell Cycle Arrest by Targeting CDC25A in Human Hepatocellular Carcinoma

**DOI:** 10.1371/journal.pone.0280394

**Published:** 2023-01-06

**Authors:** The PLOS ONE Editors

Following the publication of this article [1], concerns were raised regarding results presented in Figs 3C and 4D, and with some of the cell lines reported in this study. Specifically,

- In Fig 3D, the 5^th^ and the 7^th^ tumour presented for the control group appear similar.
- In Fig 4D, the 6^th^ tumour presented for the control group appears similar to the 3^rd^ tumour presented for the Lv-let-7c group.
- After the publication of this article [1], the cell line HepG2(no.HB-8065) reported in this study as being a Human hepatocellular carcinoma cell line, has been identified as a misclassified hepatoblastoma cell line instead [2, 3].
- After the publication of this article [1], the cell line SMMC-7721 reported in this study as being a human hepatocellular carcinoma cell line, has been identified as a contaminated HeLa derivative cell line instead [4, 5, 6].
- After the publication of this article [1], the cell line L-02 reported in this study as being a human immortalized liver cell line has been identified as a contaminated HeLa derivative cell line instead [6, 7].

The first author states that images were inadvertently duplicated during the preparation of the Fig 3C and Fig 4D results, and provided underlying data for the Fig 3 results. The first author stated that they were unable to provide a correct image for the incorrect Fig 4D Lv-let-7c panel because the tumour was so small (weighing only 0.01g) that no photo was taken. In light of the missing data, this issue cannot be fully resolved.

The first author comments that they do not agree that the cell lines are contaminated, stating that the HepG2(no.HB-8065) was obtained from ATCC [8], which indicated that “Hep G2 [HEPG2] is a cell line exhibiting epithelial-like morphology that was isolated from a hepatocellular carcinoma of a 15-year-old, White, male youth with liver cancer.”, and that the SMMC-7721 and L-02 cell lines were obtained from the Shanghai Institutes for Biological Sciences of Chinese Academy of Sciences, which provided cell line STR certificates when the cell lines were purchased. The STR certificates have not been shared with the journal for editorial review.

The authors provided individual level data underlying the Fig 4ABC results, as well as underlying data for the Western blots presented in Figs 5C, 6ABC, and Fig 7A, however not all underlying images provided shows uncropped and unaltered blots as requested by the journal. The individual level data underlying the Fig 3B results are no longer available. The first author states that all remaining underlying data remain available upon request.

In light of the concerns pertaining to the use of misidentified and contaminated cell lines, which affect the reliability of the published results and conclusions, the *PLOS ONE* Editors retract this article.

XZ agreed with the retraction and apologized for the issues with the published article. LW, JY, HJ, QW, ZY, and FW either did not respond directly or could not be reached.

## References

[1] Zhu X, Wu L, Yao J, Jiang H, Wang Q, Yang Z, et al. (2015) MicroRNA let-7c Inhibits Cell Proliferation and Induces Cell Cycle Arrest by Targeting CDC25A in Human Hepatocellular Carcinoma. PLoS ONE 10(4): e0124266. doi: 10.1371/journal.pone.0124266 25909324 PMC4409336

[2] https://www.cellosaurus.org/CVCL_0027

[3] López-Terrada D, Cheung SW, Finegold MJ, and Knowles BB (2009) Hep G2 is a hepatoblastoma-derived cell line. Human Pathology 40(10):1512–5. doi: 10.1016/j.humpath.2009.07.003 19751877

[4] https://www.cellosaurus.org/CVCL_0534

[5] Rebouissou S, Zucman-Rossi J, Moreau R, Qiu Z, and Hui L (2017) Note of caution: Contaminations of hepatocellular cell lines. Journal of Hepatology 67(5): 896–897. doi: 10.1016/j.jhep.2017.08.002 28807831

[6] Ye F, Chen C, Qin J, Liu J. and Zheng C (2015), Genetic profiling reveals an alarming rate of cross-contamination among human cell lines used in China. The FASEB Journal, 29: 4268–4272. doi: 10.1096/fj.14-266718 26116706

[7] https://www.cellosaurus.org/CVCL_6926

[8] https://www.atcc.org/products/hb-8065

